# CORRELATION BETWEEN TYPES OF MINDSET AND QUALITY OF LIFE EVALUATION IN PATIENTS WITH SCOLIOSIS

**DOI:** 10.1590/1413-785220233105e266234

**Published:** 2023-12-18

**Authors:** WESLEY WILIAN COSTA MARTINS, LEONARDO SARDAS, RODOLFO GUEDES PEREIRA NUNES BARBOSA, RODRIGO GÓES MEDÉA DE MENDONÇA, ALBERTO GOTFRYD, MARIA FERNANDA SILBER CAFFARO, PATRÍCIA MARIA DE MORAES BARROS FUCS, ROBERT MEVES

**Affiliations:** 1 Irmandade da Santa Casa de Misericordia de Sao Paulo, Department of Orthopedics and Traumatology “Pavilhao Fernandinho Simonsen”, Sao Paulo, SP, Brazil; 2 Irmandade da Santa Casa de Misericordia de Sao Paulo, Departamento de Ortopedia e Traumatologia “Pavilhao Fernandinho Simonsen”, Spine Surgery Group, Sao Paulo, SP, Brazil

**Keywords:** Scoliosis, Spine, Quality of Life, Behavior, Patient Health Questionnaire, Escoliose, Coluna Vertebral, Qualidade de Vida, Comportamento, Questionário de Saúde do Paciente

## Abstract

Scoliosis is a pathology with multiple etiologies that leads to aesthetic changes, increased morbidity and, especially, psychological damage.

**Objective:**

This work aims to compare two mindset types (fixed and growth) and assess levels of quality of life in individuals with scoliosis.

**Methods:**

Two questionnaires, Scoliosis Research Society-30 (SRS-30) and Early-Onset Scoliosis-24 Questionnaire (EOSQ-24), associated with the “Health Mindset Scale,” were used. We applied the SRS-30 to patients who were independent or whose diagnosis of spinal deformity occurred after the age of 10 years. For patients diagnosed before the age of 10 or who presented dependence due to cognitive impairment, caregivers were subjected to the “Health Mindset Scale” and EOSQ-24 questionnaires.

**Results:**

The sample consisted of 35 patients aged from 4 to 46 years, the majority aged from 15 to 18 years old (42.9%), female (71.4%), and with neuromuscular scoliosis (28.6%). The only significant result (p = 0.060) was the increase in pain/discomfort scores in the EOSQ-24 for a patient with a growth mindset. Lastly, there was no statistical difference between groups, however, in patients with a growth mindset, there was a tendency (p = 0.060) to have a higher pain/discomfort score, assessed via the EOSQ-24 score, reported by the caregiver. ***Level of Evidence III, Retrospective Comparative Study.***

## RESUMO

A escoliose é uma patologia com múltiplas etiologias e que acarreta alterações estéticas, aumento de morbidade e principalmente danos psicológicos.

**Objetivo:**

Comparar dois tipos de mindset (fixo e construtivo) e o nível de qualidade de vida.

**Métodos:**

Foram utilizados dois questionários, o Scoliosis Research Society-30 (SRS-30) e o Early-Onset Scoliosis-24 Questionnaire (EOSQ-24), associados à escala Health Mindset Scale. Aplicamos o SRS-30 em pacientes independentes ou cujo diagnóstico de deformidade na coluna ocorreu após os 10 anos. Já no caso de pacientes com diagnóstico antes dos 10 anos ou que apresentassem dependência devido a dificuldades cognitivas, os cuidadores foram submetidos à Health Mindset Scale e ao EOSQ-24.

**Resultados:**

A amostra foi composta por 35 pacientes com idades entre 4 e 46 anos, sendo a maioria entre 15 e 18 anos (42,9%), do sexo feminino (71,4%) e com escoliose do tipo neuromuscular (28,6%). O único resultado com significância (p = 0,060) foi o aumento dos escores de dor/desconforto nos questionários EOSQ-24 em paciente com mindset de crescimento. Por fim, não houve diferença estatisticamente significante entre os grupos, porém, em pacientes com mindset construtivo, houve tendência (p = 0,060) de maior escore de dor/desconforto avaliado por meio do EOSQ-24 e referido pelo cuidador. **Nível de Evidência III, Estudo Retrospectivo Comparativo.**

## INTRODUCTION

Scoliosis is defined as a deformity with a 3D deviation of the spine.^1^ Based on its etiology, it can be divided into four very distinct groups: neuromuscular; syndromic; congenital; and idiopathic. ^(^ ^2^ ^),(^ ^3^ Regardless of the etiology, this comorbidity is generally associated with body changes, long-term morbidity, and significant psychological damage, which means it can be considered a psychosocial challenge for patients and caregivers. ^(^ ^4^ ^),(^ ^5^ In this context, patient-centered questionnaires are important for treatment evaluation, care protocols and definition of policies by paying entities. ^(^ ^6^

The Scoliosis Research Society-30 (SRS-30) questionnaire, modified by Asher et al. from the original questionnaire created by Haher et al., ^(^ ^7^ which proved, through internal consistency, planned score distribution and confidence level, to be an appropriate instrument for patients with scoliosis, measuring patients’ quality of life and the outcome of surgical procedures in the spine. Oliveira, Meves and Avanzi^8^ translated and validated this questionnaire for its application in Brazil. They presented a final translated version of the SRS-30 questionnaire after testing it with 20 patients, clarified how it should be scored and suggested that, in Brazil, the completion of the questionnaire should be assisted by a professional, preferably a health professional, since some patients had difficulty understanding the questions.

In addition to this questionnaire, we can use the Early-Onset Scoliosis-24 Questionnaire (EOSQ-24), developed by Corona et al., ^(^ ^9^ in the United States, and applied to caregivers of children with early-onset scoliosis (EOS), that is, scoliosis with onset before the age of 10 years. This questionnaire consists of 24 items, with 11 domains designed to assess the quality of life of children with EOS and the burden of care on their caregivers. It also has a translated and validated version for the Brazilian population, which presents excellent reliability for the application to patients with EOS, as presented by Mendonça et al. ^(^ ^6^

New ways of classifying and indicating treatments for this type of comorbidity find, in social psychology, the concept of “mindset” (an individual’s assumption about the source of their own capacity), which has recently been applied to healthcare in the United States and was formulated by Dweck in 2006. ^(^ ^10^ There are two divergent types of mindsets that fundamentally change how individuals respond to similar circumstances: the “fixed” mindset and the “growth” mindset. The “fixed” mindset is the belief that attribute is essentially immutable, and the “growth” or “constructive” mindset is the belief that this attribute can be improved through consistent effort. This research on mindset and its potential to influence behavioral outcomes was conducted and validated for the first time in the realm of intelligence, specifically on children attending school. It was observed that the growth mindset was associated with better performance and with the tendency to seek new challenges. Furthermore, simple interventions to promote constructive mindset have been shown to improve both performance during classes and students’ grades. ^(^ ^10^

The mindset theory has recently been applied to the medical field, also in the United States, through a questionnaire formulated with four questions and with answers ranging from 1-6, in which “1” would be to completely agree and “6” to completely disagree. According to the final score, individuals were then divided into two groups: fixed or constructive mindsets. ^(^ ^10^ ^)-(^ ^12^ While individuals with a “constructive” mindset tended to see health as something that could be improved through their behaviors, those with a “fixed” mindset regarded health as something immutable. This was observed through contrasting responses to the disease in terms of behaviors and treatment outcomes. It has been found that constructive-minded patients typically have better adaptive responses to their diseases, both in cases in which they were previously healthy and in cases of chronic diseases. ^(^ ^12^

Postoperative patients with constructive mindsets consistently present lower scores on pain scales, as in cases of tonsillectomy and pectus excavatum corrections. ^(^ ^13^ ^),(^ ^14^ In the case of chronic diseases, patients with diabetes bearing this mindset present better glycemic control, and constructive-minded individuals who receive renal transplant show better quality of life. ^(^ ^15^ ^),(^ ^16^ For healthy individuals, constructive mindset is associated with better eating habits and physical activity, both in eutrophic individuals and those with obesity. ^(^ ^17^ ^).(^ ^18^

Given the effects of the mindset on various health areas, we applied this 4-question questionnaire, that is, the “Health mindset scale” after translation into Brazilian Portuguese and cross-cultural validation, ^(^ ^19^ to patients with spinal deformities, comparing rates of quality of life, which will be measured through the SRS-30 and EOSQ-24 questionnaires. The hypothesis was that patients with constructive mindsets would report a higher quality of life than patients with fixed mindsets.

## METHODS

The study took place in a tertiary hospital located in the capital city of the state of São Paulo, with the approval of the Research Ethics Committee of the Irmandade de Misericórdia da Santa Casa de São Paulo (Opinion No. 5,114,313).

All patients and caregivers who participated in the study were adequately informed and signed an informed consent form, which included appropriate specifications about the study and the role of the participant.

The “Health Mindset Scale”-translated into Brazilian Portuguese, according to the international guideline for cross-cultural adaptation-was used. ^(^ ^20^ Along with the “Health Mindset Scale,” the SRS-30 was applied to independent patients and to those whose spinal deformity diagnosis occurred after the age of 10 years. In the case of patients diagnosed before the age of 10 years or who presented dependence due to cognitive impairment, the “Health Mindset Scale” and EOSQ-24 questionnaires were answered by the caregivers. The evaluators contacted patients both in person, during outpatient visits, and by telephone call, for data collection. Subsequently, results were compared to evaluate the profile of the groups studied.

### Statistical analysis

Continuous variables are expressed as mean, standard deviation, median, and interquartile range. The categorical variables, in turn, are expressed by their absolute number of occurrence and their percentages. For internal consistency analysis, Cronbach’s alpha reliability test was used in each group of questions that characterized a questionnaire domain, in addition to the global internal consistency index involving the entire questionnaire. For the analysis of the ceiling and floor effects, it was considered that 15% of patients who obtained the lowest or the highest possible score determined the effect. Data analyses were performed using the SPSS 23.0 program for MAC (IBM SPSS Inc., Chicago, IL). A p < 0.05 value was considered statistically significant.

For discriminative validity, comparisons between categorical variables were performed using non-parametric tests (Kruskar-Wallis and Mann Whitney U) and Spearman’s correlation coefficients were used for continuous variables.

## RESULTS

The translated and cross-culturally adapted questionnaires were applied to the patients included in the study. The sample consisted of 35 patients aged from 4 to 46 years(M = 15.48; SD = 7.12), most of them being aged from 15 to 18 years (42.9%), female (71.4%), and with neuromuscular scoliosis (28.6%). Table 1 presents details on the profile of the sample regarding gender, age group, and type of scoliosis.

**Table 1:** Sample profile.

Characteristic	f	%
Sex		
Female	25	71.4
Male	10	28.6
Age group		
4 to 10 years	6	17.1
11 to 14 years	9	25.7
15 to 18 years	15	42.9
Over 18 years	5	14.3
Type of Scoliosis		
Spinal cord abnormality	1	2.9
Congenital or structural	7	20.0
Idiopathic	7	20.0
Neuromuscular	10	28.6
Syndromic	4	11.4
Missing information	6	17.1

### Internal consistency of the instruments

#### Internal consistency of the Health Mindset Scale

The internal consistency of the three items was satisfactory (α = 0.723). Table 2 presents descriptive statistics for each item and α for excluded items.

**Table 2:** Descriptive statistics and α if the item is excluded from the Health Mindset Scale.

Item	M	SD	α if the item is deleted
1. Your body has a defined health condition or level and you cannot do much to change that.	3.68	1.77	0.75
2. You cannot quite change your health.	4.22	1.61	0.45
3. You can try to feel better, but you cannot change your health.	4.74	1.52	0.68

M = mean; SD = standard deviation.

In addition, Table 3 presents bivariate inter-item correlations.

**Table 3:** Inter-item correlations and item-total of the Health Mindset Scale.

Item	1.	2.	3	Item-total correlation
1. Your body has a defined health condition and you cannot do much to change that.	1			0.45
2. You cannot quite change your health.	0.51	1		0.69
3. You can try to feel better, but you cannot change your health.	0.29	0.60	1	0.50

Strong correlations were observed between items 1 and 2 and between items 2 and 3. However, a poor correlation was observed between items 1 and 3. Item-total correlations ranged from 0.45 (item 1) to 0.69 (item 2).

#### Internal Consistency of SRS-30

Table 4 shows the internal consistency of each dimension of the SRS-30.

**Table 4:** Cronbach’s alpha coefficients of the Scoliosis Research Society-30 (SRS-30) domains.

Domain	α
Function/Activity	0.54
Pain	0.85
Self-Image/Appearance	0.61
Mental Health	0.50
Satisfaction with Management	0.80

The alphas ranged from 0.50 (mental health) to 0.85 (pain). Two dimensions presented a coefficient below recommendations (Function/Activity and Mental Health).

#### Internal Consistency of EOSQ-24

Table 5 presents the internal consistency of each EOSQ-24 dimension.

**Table 5:** Cronbach’s alpha coefficients of the Early-Onset Scoliosis-24 Questionnaire (EOSQ-24) domains.

Domain	α
General Health	0.26
Pain/Discomfort	0.78
Pulmonary Function	0.08
Transfer	Singular Item
Physical Function	0.74
Daily Living	0.44
Fatigue/Energy Levels	0.42
Emotion	0.51
Parental Impact	0.57
Financial Impact	Singular Item
Satisfaction	0.77

Coefficients ranged from 0.08 (Pulmonary Function) to 0.78 (Pain/Discomfort). Six dimensions presented alphas below recommendations (< 0.60).

Bivariate correlations between scores on the Health Mindset Scale and SRS-30 (Table 6), and between scores on the Health Mindset Scale and EOSQ-24 (Table 7) are presented below:

**Table 6:** Correlation coefficients between the Health Mindset Scale and Scoliosis Research Society-30 (SRS-30).

Domain	Spearman’s Rho	p-value
Function/Activity	− -−0.16	0.512
Pain	− -−0.17	0.501
Self-Image/Appearance	0.16	0.515
Mental Health	0.07	0.775
Satisfaction with Management	−− -−0.009	0.972

**Table 7:** Correlation coefficients between the Health Mindset Scale and Early-Onset Scoliosis-24 Questionnaire (EOSQ-24).

Domain	Spearman's Rho	p-value
General Health	0.04	0.832
Pain/Discomfort	0.44*	0.034
Pulmonary Function	-−0.07	0.746
Transfer	0.02	0.900
Physical Function	0.09	0.654
Daily Livings	-−0.25	0.247
Fatigue/Energy Levels	-−0.14	0.515
Emotion	-−0.18	0.402
Parental Impact	-−0.05	0.810
Financial Impact	-−0.14	0.515
Satisfaction	0.05	0.790

* Significant correlation (p < 0.05).

The Pain/Discomfort score of EOSQ-24 was significant and the Health Mindset Scale score was moderate and positive. That is, the higher the pain score on this scale, the greater its disparity with the items of the Health Mindset Scale.

#### Comparison between SRS-30 and EOSQ-24 by types of mindset types

Mann-Whitney tests were performed to compare SRS-30 and EOSQ-24 scores by types of mindset (Table 8).

**Table 8:** Non-parametric comparison between the Scoliosis Research Society-30 (SRS-30) and Early-Onset Scoliosis-24 Questionnaire (EOSQ-24) scores, considering differences between fixed and growth mindset types.

Domain	Fixed	Growth	p-value Mann-Whitney
SRS-30			
Function/Activity	3.00 0.54	3.10 0.54	0.798
Pain	3.60 1.48	3.55 0.98	0.798
Self-Image/Appearance	3.55 0.52	3.47 0.65	0.721
Mental Health	3.68 0.84	3.40 0.58	0.574
Satisfaction with management	3.25 0.64	3.55 1.07	0.442
EOSQ-24			
General Health	56.25 13.11	61.02 13.89	0.443
Pain/Discomfort	39.58 22.93	65.44 25.59	0.060
Pulmonary Function	79.16 23.27	72.79 21.75	0.450
Transfer	62.50 30.61	70.58 26.86	0.533
Physical Function	62.48 30.62	63.22 32.28	0.832
Daily Living	72.91 22.93	53.67 38.72	0.254
Fatigue/Energy Levels	62.50 28.50	61.76 23.58	0.943
Emotion	64.58 25.51	51.47 28.25	0.394
Parental Impact	55.83 23.54	61.47 17.02	0.698
Financial Impact	58.33 25.81	54.41 23.77	0.881
Satisfaction	50.00 27.38	57.35 24.62	0.428

Mean ± standard deviation.

The results indicated only a marginally significant difference in the pain/discomfort score in the EOSQ-24 (p = 0.060), indicating that patients with a growth mindset scored higher in this dimension.

## DISCUSSION

Scoliosis is a condition that limits the daily living of those who suffer from it, causing a relevant impact on their quality of life and that of their caregivers^6^. In our study, we obtained a sample of 35 patients aged from 4 to 46 years (M = 15.48; SD = 7.12), with most individuals aged from 15 to 18 years (42.9%), female (71.4%), and with neuromuscular scoliosis (28.6%).^11^

Satisfactory internal consistency was observed for the three items (α = 0.723) of the “Health mindset scale.” Strong correlations were observed between items 1 and 2 and between items 2 and 3. However, poor correlation was observed between items 1 and 3. Item-total correlations ranged from 0.45 (item 1) to 0.69 (item 2), as observed in the translation of the scale into Brazilian Portuguese. ^(^ ^20^

The pain/discomfort scores were significant (p = 0.060) for the EOSQ-24 questionnaires in patients with a growth mindset. This finding contradicts the literature-for example, Joseph et al. ^(^ ^11^- since constructive-minded patients are expected to obtain lower pain perception scores. The reason for this incongruity may be related to the fact that answers were given by caregivers, therefore, their perceptions of their own fatigues, emotional impacts and economic burdens might have been reflected in responses.

There was no statistically significant difference between patients with different mindset types and the SRS-30 and EOSQ-24 quality of life scores (8 and 27 patients, respectively). As a limitation of this study, we mention the difficulty of patients to accurately understand the concepts of growth mindset, even though such concepts were explained during the questionnaires application. Patients who attend to outpatient clinics in our service generally have low socioeconomic and educational levels. It was noteworthy that the interviewees needed further clarification about what was being requested to understand the context of statements or assign them scores: we observed that some individuals gave opposite evaluations (agree or disagree) to statements with similar contexts. Moreover, some caregivers/patients were concerned about the negative influence of their responses on the treatment, especially when they agreed with statements that corresponded to the fixed mindset. This apprehension continued even after the interviewer explained that the results of the questionnaires would not interfere in future follow-up. These factors may have influenced responses and results.

A study by Joseph et al., ^(^ ^11^ in which 110 individuals were evaluated-mostly women (85.5%), mean age of 13.1 ± 1.4 years, participants of a program for treatment with orthosis, and who were able to complete the questionnaire with a good understanding- demonstrated that patients with growth mindsets generally had greater well-being than those with fixed mindsets, especially during treatment, and accepted such treatment better. In a study by Krain et al., ^(^ ^13^ 1,005 caregivers and their children were interviewed during the postoperative periods of tonsillectomy and adenoidectomy. The study population had no significant difference between genders, a mean age of 6.24 ± 2.93 years with, respectively, 72% and 79.9% of mothers and fathers, and these had attended school at least up to high school. Results demonstrated that caregivers with fixed mindsets reported higher pain scores and greater use of painkillers in the recent postoperative period, even though the pain scores were not significantly different between patients.

Comparing both studies to ours, we observed that these maintained a single type of treatment, only interviewed participants before the use of the orthosis and in the recent postoperative period, and had larger and more homogeneous samples. Meanwhile, our study included different types of scoliosis, treatments and stages (surgical or not, pre- and post-medical intervention) and various age groups, producing a more heterogeneous sample. It is also important to indicate that the total number of patients in our study can also be considered as a limiting factor (35 patients). These variables may have impacted the results found, which differ from the reviewed literature. ^(^ ^21^

## CONCLUSION

The application of the mindset scale divided subjects into two groups: fixed and constructive mindset (8 and 27 patients, respectively) The correlation showed no statistical difference between the groups, however, a higher pain/discomfort score tended (p = 0.060) to be found in patients with a constructive mindset, as assessed by the EOSQ-24 through caregivers’ reports.

This study opens a new perspective in the understanding of the referred capacity of psychometric questionnaires and their dependency toward patients or caregivers. However, our study still requires further development: changes such as increasing the study sample and selecting a homogeneous population to be evaluated are necessary.
